# Effective electrochemical trichloroethylene removal from water enabled by selective molecular catalysis

**DOI:** 10.26599/cf.2024.9200015

**Published:** 2024-09

**Authors:** Yuanzuo Gao, Wanyu Zhang, Chungseok Choi, Bo Shang, Seonjeong Cheon, Aidan Francis Meese, Jae-Hong Kim, Donghui Long, John Fortner, Hailiang Wang

**Affiliations:** 1 Department of Chemistry, Yale University, New Haven, CT 06520, USA; 2 Energy Sciences Institute, Yale University, West Haven, CT 06516, USA; 3 Department of Chemical and Environmental Engineering, Yale University, New Haven, CT 06520, USA; 4 State Key Laboratory of Chemical Engineering, East China University of Science and Technology, Shanghai 200237, China

**Keywords:** electrocatalysis, electrified membrane, trichloroethylene, water treatment

## Abstract

Electrochemistry can provide a viable and sustainable way to treat water polluted by chlorinated volatile organic compounds. However, the removal and valorization of trichloroethylene (TCE) remains as a challenge due to the lack of suitable electrocatalysts with high selectivity and activity. We herein present a catalyst, comprising cobalt phthalocyanine (CoPc) molecules assembled onto multiwalled carbon nanotubes (CNTs), that can electrochemically decompose aqueously dissolved TCE into ethylene and chloride ions at record high rates with close to 100% Faradaic efficiency. Kinetics studies reveal that the rate-determining step is the first electron transfer without proton involvement. We further show that replacing the CNT support with reduced graphene oxide (rGO) can improve the TCE treatment efficacy because of the two-dimensional nanostructure of rGO and its stronger interaction with CoPc molecules. Incorporating the CoPc/rGO catalyst into an electrified membrane filtration device, we demonstrate 95% TCE removal from simulated water samples with environmentally relevant TCE and electrolyte concentrations.

## Graphical Abstract

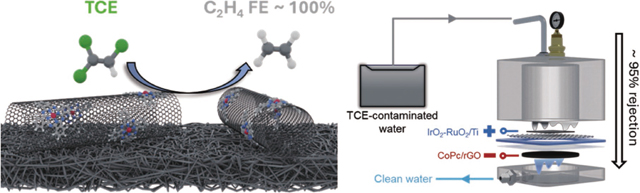

## 1 Introduction

Trichloroethylene (TCE) is one of the most widely used chlorinated volatile organic compounds (VOCs) in industry. It is extensively used as a solvent or cleaning agent in refrigerants, dry cleaning, and metal and electronic degreasing^1, 2^. About 225 million pounds of TCE is produced annually^3, 4^. TCE is prone to spreading into the environment through improper handling or disposal, and it can form a dense non-aqueous liquid phase in the underground^5–7^. With its long lifetime in the natural environment, TCE ubiquitously distributes in groundwater systems across the world^8–11^.

Unfortunately, TCE is highly toxic. Exposure to TCE can cause severe damage to various critical organs and systems in the human body or even cancer^12, 13^. Bioremediation is one of the first technologies developed to treat environmental TCE^14^. However, this method is limited by long degradation time, susceptibility to influences from other electron acceptors in the reaction matrix^15, 16^, and incomplete decomposition leading to partially dechlorinated products which can be more toxic than TCE^17–19^. Chemical remediation, on the other hand, can afford much higher reaction rates and more selective electron transfer^20^. For instance, zerovalent iron, boron-doped iron, and chemically reduced cobalamin all exhibit the capacity to remove about 75% of dissolved TCE within 10 days^20–23^. Nevertheless, this method still faces the drawbacks of incomplete dechlorination, surface passivation, and the need for strong chemical reductants.

Electrochemical decomposition, potentially powered by renewable energy, emerges as an effective and sustainable strategy for TCE removal and valorization. Various materials including metals and metal complexes have been studied for this purpose ^15, 24–29^. For example, Ag can achieve close to 100% TCE removal, but a large overpotential is required, which also promotes the competing hydrogen evolution reaction (HER), resulting in a 92% loss in current efficiency^24^. A Pd-Fe alloy can degrade TCE with Faradaic efficiency (FE) up to 90%, yet its production rate of ethylene is only about 0.001 mmol·g^−1^·s^−1^, while toxic, partially dechlorinated products such as dichloroethylene and vinyl chloride persist^25^. Iron embedded in nitrogen-doped carbon has shown complete conversion of TCE to ethylene and ethane at 0.007 mmol·g^−1^·s^−1^ with a 50% FE^26^. Molecular electrocatalysts have also been explored for TCE remediation, but their performance is still far from ideal. Manganese phthalocyanine (MnPc) and Hemin can only catalyze incomplete TCE dechlorination with FE up to 15% ^27, 28^. In our prior work, we discovered that cobalt phthalocyanine molecules supported on multiwalled carbon nanotubes (CoPc/CNT) can catalyze the electrochemical reduction of dichloroethane (DCA) into ethylene with high FE and reaction rates^30^. The outstanding performance was hypothesized to arise from the intrinsic activity of the molecular catalyst as well as the high surface area and electrical conductivity of the CNT support, which can potentially be beneficial to the decomposition of other chlorinated VOCs.

In this work, we studied CoPc/CNT for electrochemical TCE decomposition into ethylene and chloride in aqueous environment. In a 0.1 M KHCO_3_ electrolyte containing ~ 9 mM TCE, CoPc/CNT exhibits an onset potential of approximately −0.48 V (all potentials in this work are referenced to the reversible hydrogen electrode, RHE, unless otherwise specified) and reaches a turnover frequency (TOF) of ~ 1.5 s^−1^ at −0.68 V. The FE towards ethylene is close to unity in this potential window and partially dechlorinated products are not detected. Kinetic measurements indicate that the rate-determining step is the first electron transfer without proton involvement. To further enhance the feasibility of our catalyst in practical water treatment, CoPc molecules are incorporated into an electrified membrane based on reduced graphene oxide (rGO). The membrane filtration device can achieve and maintain > 95% TCE removal from simulated ground water samples in an application-relevant flow-through mode.

## 2 Results and discussion

**Fig. 1.**
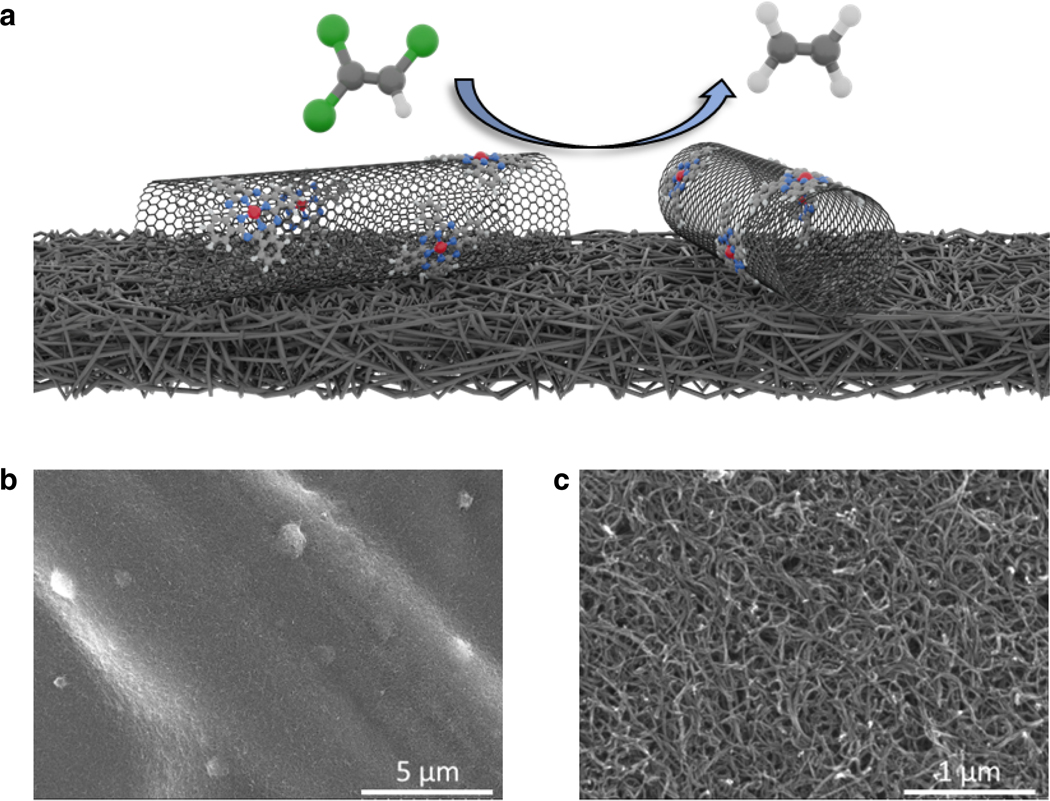
Schematic illustration and Morphology. **a–c,** Schematic illustration (a) and SEM images (b and c) of CoPc/CNT catalyst drop-casted on carbon fiber paper for TCE dechlorination.

**Fig. 2.**
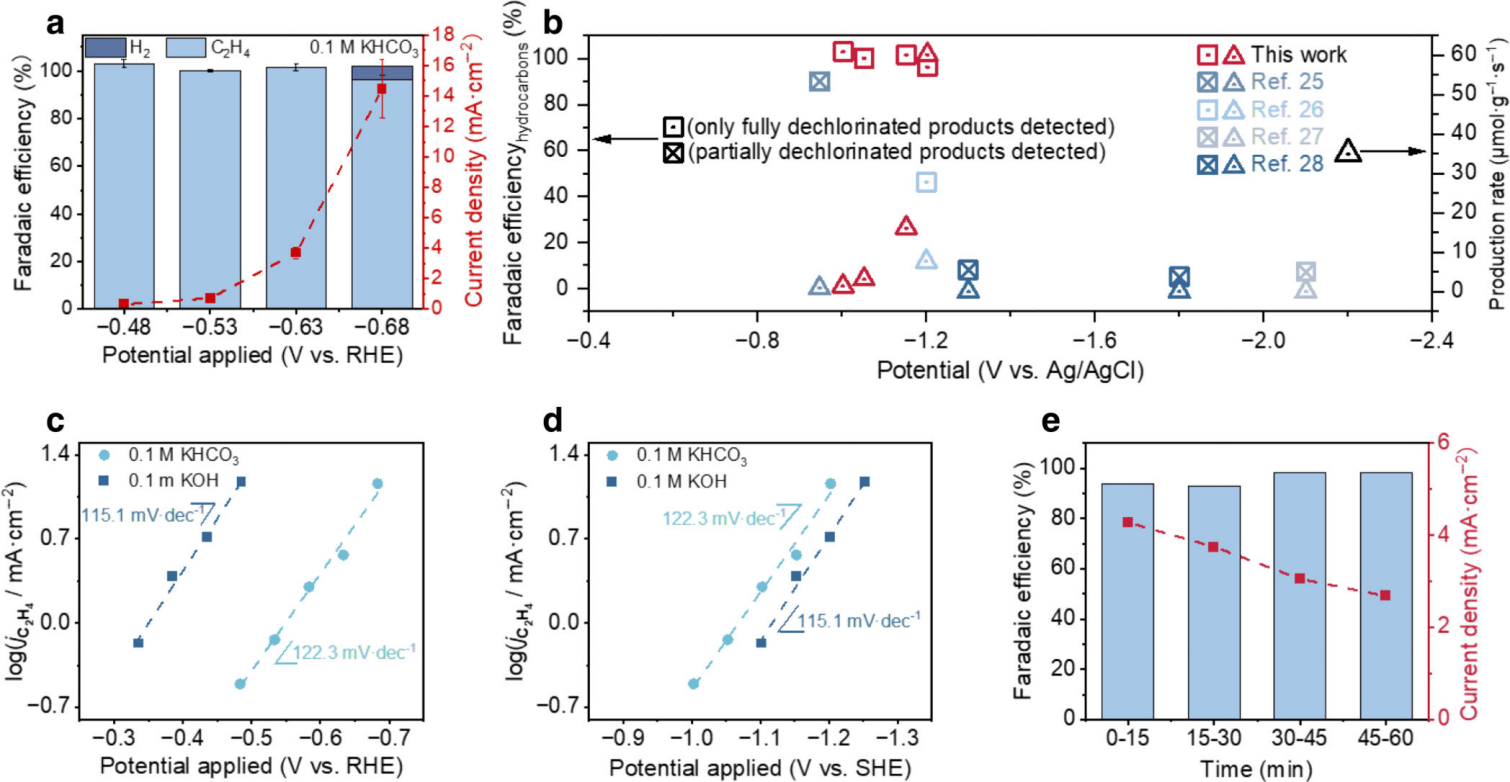
Electrocatalytic performance. **a**, Electrochemical TCE dechlorination performance (FE and total current density) of CoPc/CNT in 0.1 M KHCO_3_ solution. **b**, Comparison of TCE dechlorination selectivity and production rate for ethylene between CoPc/CNT and other catalysts reported to date. **c**, **d**, Tafel curves for ethylene formation from TCE reduction catalyzed by CoPc/CNT at different pH values on RHE (c) and SHE scales (d). **e**, Stability of TCE dechlorination catalyzed by CoPc/CNT at −0.64 V in 0.1 M KHCO_3_. Error bars in (a) represent standard deviation from 3 independent measurements.

The CoPc/CNT catalyst material was prepared following our prior work and drop-casted onto carbon fiber paper (CFP) to form catalytic electrodes (Fig. 1)^30^. The cyclic voltammogram of the as-prepared electrode exhibits the characteristic redox feature of CoPc molecules (Supplementary Fig. S1)^31^, which indicates successful anchoring of CoPc molecules on CNT surfaces and good electrical contact between these two components^32^. The catalytic performance for TCE dechlorination was studied in a gas-tight H-cell with TCE vapor being continuously carried into the electrolyte by Ar under 700 rpm stirring to stabilize the reactant concentration (~ 9 mM) and mass transport conditions. In 0.1 M aqueous KHCO_3_ (pH = 8.8), CoPc/CNT catalyzed TCE dechlorination with close to 100% FE_ethylene_ in the potential range from −0.48 to −0.68 V (Fig. 2a). At −0.68 V (−1.4 V vs. Ag/AgCl), a current density of ~ 15 mA·cm^−2^ with an FE_ethylene_ as high as 95% was achieved, while no partially dechlorinated products such as dichloroethylene and vinyl chloride were detected (Supplementary Fig. S2). When normalizing this reaction rate to the total mass of CoPc/CNT catalyst, a production rate of 60 μmol·g^−1^·s^−1^ for TCE decomposition to ethylene is obtained, which is ~ 10 times higher than the other reported catalysts at similar or even more negative potentials (Fig. 2b). The high selectivity for complete TCE dechlorination, namely the absence of harmful dichloroethylene and vinyl chloride byproducts, is another advantage of our catalyst compared to the others (Fig. 2b).

Tafel analysis for CoPc/CNT-catalyzed TCE dechlorination to ethylene was conducted in 0.1 M aqueous KHCO_3_ and KOH solutions with different pH values. The catalysis in the KOH electrolyte also exhibited high selectivity for ethylene (Supplementary Fig. S3). A Tafel slope around 120 mV·dec^−1^ with high linearity was observed in both conditions (Figs. 2c and 2d), which indicates a rate-determining first electron transfer step with the symmetry factor assumed to be 0.5^33^. On the RHE scale, the two Tafel curves separate by ~ 50 mV per pH unit while they largely overlap on the standard hydrogen electrode (SHE) scale (Figs. 2c and 2d). The slight deviation from the ideal pH dependence of ~ 60 mV per pH unit is likely attributed to experimental errors. This result suggests that electrochemical TCE dechlorination to ethylene catalyzed by CoPc/CNT is mostly a pH-independent process, consistent with our prior work on CoPc-catalyzed DCA dechlorination^30^. The absence of proton participation in the rate-determining step, unlike similar reactions over metal surfaces^15, 25, 34^, could be a direct result of the molecular catalyst’s single-site nature and play a key role in suppressing HER and consequently promoting TCE dechlorination^30^.

Despite the unprecedented catalytic activity and selectivity for TCE dechlorination, CoPc/CNT is subject to inferior durability under reaction conditions. After an hour of electrolysis at −0.64 V, although the selectivity towards ethylene remained close to 100%, the partial current density decreased to ~ 65% of the original level (Fig. 2e). Current decay was found to be more severe at more negative potentials (Supplementary Fig. S4). The cyclic voltammogram obtained from the used electrode lacks the characteristic Co^I^/Co^II^ redox feature (Supplementary Fig. S1), implying deactivation of the CoPc site. To test whether the catalytic site can be reactivated by cathodic or anodic treatment, used CoPc/CNT electrodes were held at various reductive or oxidative potentials in clean 0.1 M KHCO_3_ solutions, which, nevertheless, did not recover the redox feature of the Co center (Supplementary Fig. S5). Oxidative steps introduced during electrolysis did not alleviate current decay but worsened ethylene selectivity (Supplementary Fig. S6). We also looked into the electrolyte effect. Neither phosphate buffer nor CsHCO_3_ was able to stabilize the catalyst’s activity towards ethylene formation (Supplementary Fig. S7). These results indicate that the CoPc structure might have been permanently damaged in the deactivation.

To test the hypothesis, Raman spectroscopy was conducted. Raman spectra of as-prepared CoPc/CNT and CoPc/CNT after catalyzing HER at −0.64 V in 0.1 M KHCO_3_ both exhibit characteristic Co–N and C=N–C vibrations at 594 cm^−1^ and 1532 cm^−1^, respectively, same as the spectrum of pure CoPc powder (Supplementary Fig. S8) ^35, 36^. In contrast, for the used catalyst after TCE dechlorination electrolysis, the Co–N peak became essentially invisible and the C=N–C peak was severely attenuated (Supplementary Fig. S8b), indicating structural damage to the CoPc molecules. Similar changes in Raman spectra have been observed for iron phthalocyanine catalyzing the oxygen reduction reaction where generated radical species damage the phthalocyanine ring structure and lead to degradation of the molecular catalyst^37^. When we added toluene, a known hydrogen atom transfer reagent^38, 39^, into the CoPc/CNT-catalyzed TCE reduction reaction, ethane emerged as another hydrocarbon product (Supplementary Fig. S9). This result implies the existence of reaction intermediates with radical characteristics in the electrochemical TCE dechlorination process catalyzed by CoPc/CNT^38, 40^, which may be responsible for the observed CoPc structural damage.

**Fig. 3.**
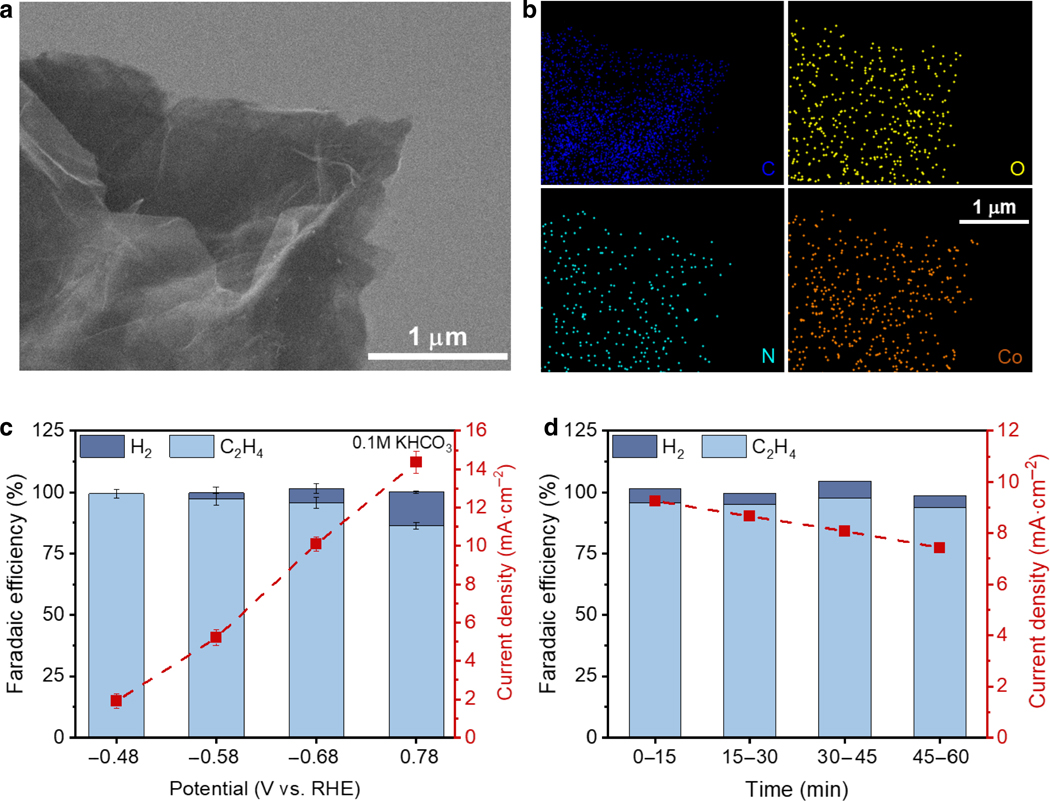
Characterization and electrocatalytic performance of CoPc/rGO. **a, b,** SEM image (a) and EDS mapping (b) of CoPc/rGO. **c**, Electrochemical dechlorination performance (FE and total current density) of CoPc/rGO in TCE-saturated 0.1 M KHCO_3_. **d**, Stability of TCE dechlorination catalyzed by CoPc/rGO at −0.68 V.

In the hope of prolonging the lifetime of CoPc-based catalysts, we chose rGO as an alternative support to CNT as it can hold approximately 2.5 times more CoPc molecules as revealed in the cyclic voltammogram (Supplementary Fig. S10). Scanning electron microscopy (SEM) and energy-dispersive X-ray spectroscopy (EDS) characterization of the CoPc/rGO hybrid material reveals a flaked structure (Fig. 3a), typical of graphene-based materials^41^, with relatively uniformly distributed CoPc molecules on the rGO surface (Fig. 3b and Supplementary Fig. S11). Electrocatalytic TCE dechlorination experiments in 0.1 M aqueous KHCO_3_ saturated with TCE showed that CoPc/rGO can also achieve selective and active TCE reduction to ethylene in the −0.48 to −0.78 V potential range (Fig. 3c). At −0.68 V, a total current density of ~ 11 mA·cm^−2^ with about 90% FE_ethylene_ was recorded, close to the performance of CoPc/CNT (Fig. 1b). To our delight, CoPc/rGO retained the high FE and close to 80% of the initial current density towards ethylene formation after an hour of continuous operation at −0.68 V (Fig. 3d). This considerably improved stability compared to CoPc/CNT, which is likely due to a higher CoPc loading on rGO than on CNTs (~ 3.2 wt.% vs. ~ 1.3 wt.%, determined from cyclic voltammetry), together with the desirable membrane formation properties of rGO^42, 43^, makes CoPc/rGO a more plausible material for electrocatalytic treatment of TCE-contaminated water.

**Fig. 4.**
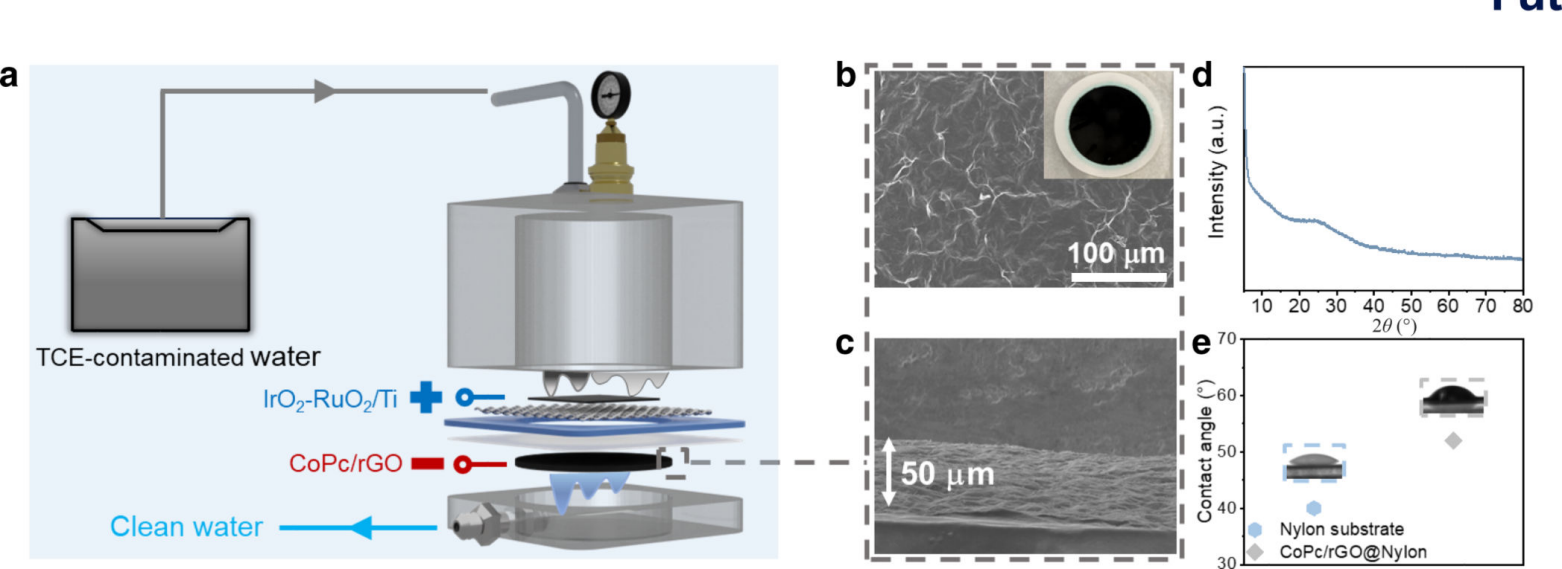
Electrified membrane filtration device. **a,** Schematic illustration of the electrified membrane filtration device. **b**, **c**, SEM images depicting top (b) and cross-sectional (c) views of CoPc/rGO@Nylon. The inset in (b) shows a photograph of a CoPc/rGO@Nylon membrane. **d**, X-ray diffraction pattern of a CoPc/rGO film. **e**, Water contact angles of CoPc/rGO@Nylon and Nylon.

To investigate the feasibility of using CoPc/rGO in removing TCE from contaminated water, we designed a flow-through electrified membrane device. As shown in Fig. 4a, this filtration device contained a feed chamber and a permeate chamber, separated by the electrochemical unit. The anode was a Ti mesh coated with RuO_2_-IrO_2_, a dimensionally stable anode commonly used for commercial applications. The cathode was a Nylon-supported CoPc/rGO (CoPc/rGO@Nylon) membrane. A 20 mM aqueous Na_2_SO_4_ solution containing ~ 1 mM of TCE (~ 131 ppm) was chosen to be the feed solution to simulate the level of contamination that is usually found in waterbodies near industrial plants as well as the naturally occurring ionic strength and sulfate levels in environmental water^44^. CoPc/rGO@Nylon was fabricated by vacuum filtrating a CoPc/rGO suspension in *N*,*N*-dimethylformamide through a clean Nylon filtration membrane (Supplementary Fig. S12) to achieve a catalyst (i.e., CoPc/rGO) loading of ~ 0.3 mg·cm^−2^, which is a suitable amount for pollutant decomposition/conversion in flow-through operations according to previous work^45^. The 50 μm thick CoPc/rGO layer on Nylon has a layered structure (Figs. 4b and 4c), with a spacing of ~ 3.6 Å determined from the position of the (002) peak in the X-ray diffraction pattern (Fig. 4d)^46^, in contrast to the interwoven CoPc/CNT layer on Nylon (Supplementary Fig. S13). This layered structure is believed to enhance contaminant rejection with the help of interlayer nanochannels^43, 47^. The CoPc/rGO@Nylon membrane also maintained a hydrophilic surface with a contact angle of 51° (Fig. 4e), which can help mitigate membrane fouling^30, 48^.

**Fig. 5.**
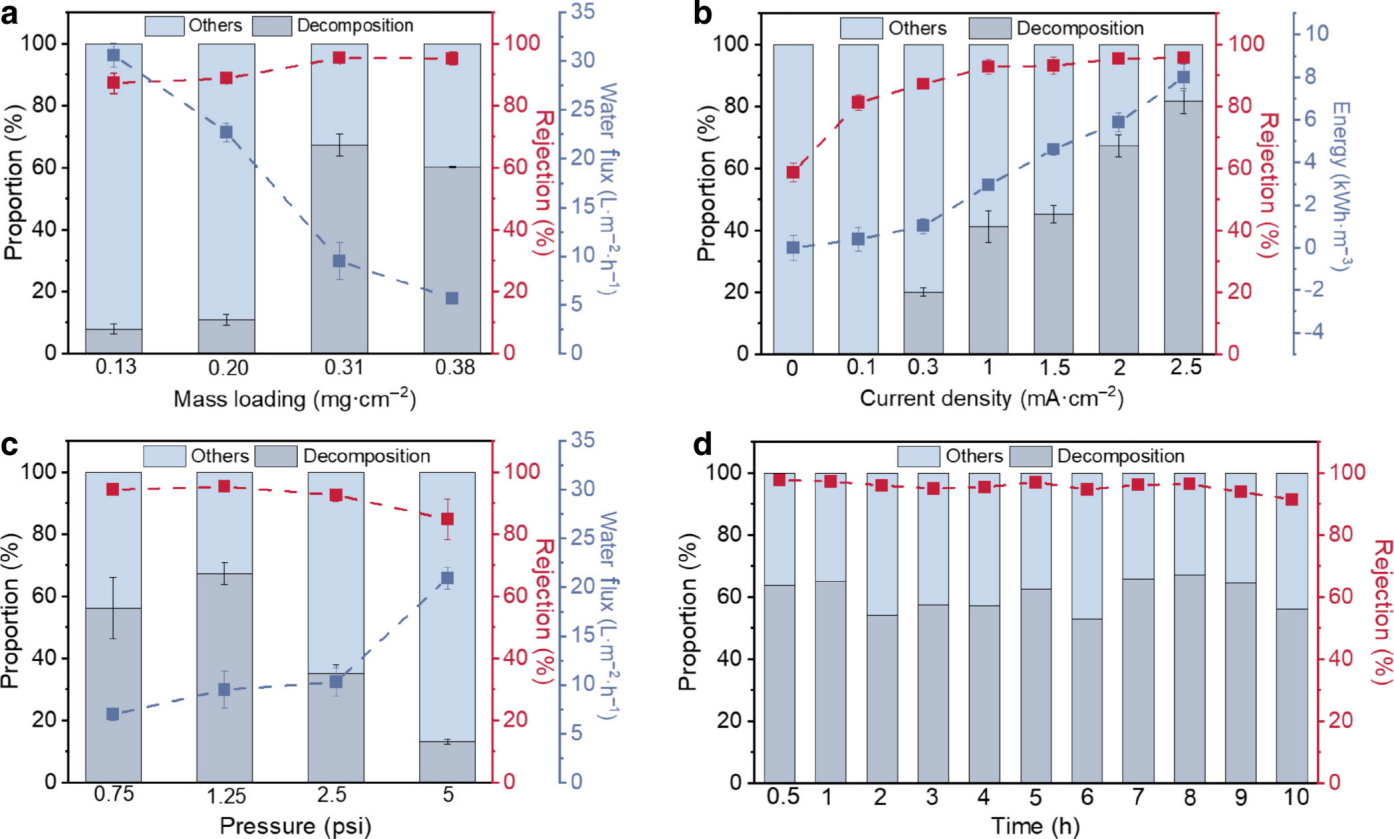
Electro-filtration of water containing 20 mM of Na_2_SO_4_ and 1 mM of TCE through CoPc/rGO@Nylon membrane. **a**, Effect of catalyst mass loading on TCE rejection, decomposition proportion, and water flux with a 2 mA·cm^−2^ operating current density and a 1.25 psi transmembrane pressure. **b**, Effect of current density on TCE rejection, decomposition portion, and energy consumption with a catalyst mass loading of 0.31 mg·cm^−2^ and a transmembrane pressure of 1.25 psi. **c**, Effect of transmembrane pressure on TCE rejection, decomposition portion, and water flux with a catalyst mass loading of 0.31 mg·cm^−2^ and a current density of 2 mA·cm^−2^. **d**, Stability of electrified filtration membrane in a 10 h operation under optimized conditions.

The electro-filtration performance was optimized by adjusting three independent parameters: catalyst mass loading, operating current density, and transmembrane pressure. First, we held the current density and transmembrane pressure at 2 mA·cm^−2^ and 1.25 psi, respectively, and adjusted the catalyst loading. Both TCE rejection and decomposition portion showed a steady increase as the catalyst loading was increased from 0.13 mg·cm^−2^. At 0.31 mg·cm^−2^, 95% rejection and 68% decomposition of TCE was achieved with a water flux of 9.5 L·m^−2^·h^−1^ (Fig. 5a). Further increase of the catalyst loading did not affect the rejection rate but lowered the TCE decomposition percentage and water flux. As control experiments, a bare Nylon membrane could reject about 50% of TCE with a 1.25 psi transmembrane pressure, but only via adsorption (Supplementary Fig. S14), whereas a rGO@Nylon membrane without CoPc, under the same condition, rejected ~ 80% of TCE with only less than 10% of the rejected TCE decomposed (Supplementary Fig. S15). This confirms the role of CoPc as the catalytic center in the electrified membrane for TCE decomposition. The rejection rate and decomposition proportion of TCE exhibited a positive relationship with the operating current density up to 2.5 mA·cm^−2^ (Fig. 5b). However, the increase of current density from 2 to 2.5 mA·cm^−2^ brought only one percentage point of improvement in rejection rate and < 17 percentage points of increase in decomposition percentage at the expense of a high energy consumption of 8 kWh·m^−3^ (Fig. 5b)^49, 50^. Thus, 2 mA·cm^−2^ was determined to be the optimal current density balancing effectiveness and energy efficiency. The transmembrane pressure was also adjusted to optimize the device performance. The best result was obtained with a transmembrane pressure of 1.25 psi (Fig. 5c).

With the optimized parameters, the electrified membrane filtration device was able to continuously operate for 10 h, with the feed solution being consumed and replenished every hour. ~ 95% TCE rejection and ~ 60% decomposition was retained throughout the entire operation (Fig. 5d). Compared to CoPc/rGO@Nylon, the Nylon-supported CoPc/CNT (CoPc/CNT@Nylon) membrane could support a considerably higher water flux of ~ 60 L·m^−2^·h^−1^ with a 0.31 mg·cm^−2^ catalyst loading and a transmembrane pressure of 1.25 psi (Supplementary Fig. S16), likely as a result of its interwoven structure (Supplementary Fig. S13). However, only ~ 70% TCE rejection and < 20% decomposition was realized at a 2 mA·cm^−2^ operating current density for CoPc/CNT@Nylon (Supplementary Fig. S16). The much better performance of CoPc/rGO@Nylon is likely because of the prolonged retention of contaminated water within the highly compact rGO layers and the ability of rGO to support more CoPc molecules which affords more catalytic sites for TCE decomposition^43, 47^. At TCE concentrations lower than 1 mM, the CoPc/rGO@Nylon membrane can achieve even higher TCE rejection and decomposition (Supplementary Fig. S17). These results demonstrate the potential of our CoPc-based materials for effective and efficient treatment for TCE contamination in groundwater.

## 3 Conclusion

In conclusion, by hybridizing CoPc molecules with CNT and rGO supports, we have developed highly selective and active electrocatalysts for treating TCE in water. These catalysts exhibit near unity FE and high current density for the electrochemical conversion of TCE into ethylene in aqueous media. Incorporation of CoPc/rGO into a dead-end electrified membrane filtration device demonstrates stable rejection of 95% of TCE from simulated environmental water samples.

## Supplementary Material

Supplemental Materials

## Data Availability

All data needed to support the conclusions in the paper are presented in the manuscript and/or the Supplementary Information. Additional data related to this paper may be requested from the corresponding author upon request.
