# Supplementary material for: Effective electrochemical trichloroethylene removal from water enabled by selective molecular catalysis
Source: Carbon Futur. Author manuscript; Available in PMC 2025 Jun 6. (PMC12143162; doi:10.26599/cf.2024.9200015)
Supplement: Supplemental Materials [file NIHMS2072960-supplement-Supplemental_Materials.pdf]

Research article

Carbon Future

Supporting Information

Effective electrochemical trichloroethylene removal from water enabled by selective molecular catalysis

Yuanzuo Gao[1,2,#], Wanyu Zhang[1,2,3,#], Chungseok Choi[1,2,†], Bo Shang[1,2], Seonjeong Cheon[1,2], Aidan Francis Meese[3], Jae-Hong Kim[3], Donghui Long[4] ✉, John Fortner[3] ✉, and Hailiang Wang[1,2] ✉

[1]*Department of Chemistry, Yale University, New Haven, CT 06520, USA*

[2]*Energy Sciences Institute, Yale University, West Haven, CT 06516, USA*

[3]*Department of Chemical and Environmental Engineering, Yale University, New Haven, CT 06520, USA*

[4]*State Key Laboratory of Chemical Engineering, East China University of Science and Technology, Shanghai 200237, China*

[†]*Present address: SKKU Advanced Institute of Nanotechnology and Department of Nanoengineering, Sungkyunkwan University, Suwon 16419, Republic of Korea*

[#]*Yuanzuo Gao and Wanyu Zhang contributed equally to this work*

Supporting information to https://doi.org/10.26599/CF.2024.9200015

In the format provided by the authors and unedited

Methods

Materials

TCE (anhydrous, 99.8%), *N*,*N*-dimethylformamide (DMF; anhydrous, 99.8%), cobalt (II) phthalocyanine (CoPc), $KHCO_3$ (99.7%), and Na_2SO_4 (99.7%) were purchased from Sigma-Aldrich. K_2SO_4 (99%) was purchased from Alfa Aesar. Multiwalled CNTs were purchased from C-Nano (product no. FT 9100). Ar (99.999%) was purchased from Airgas. Deionized water (18.2 MΩ cm at 25 °C) from a Millipore water purification system was used throughout the experiments. Unless otherwise noted, all the commercial chemicals were used without purification.

Characterization

SEM imaging and EDS mapping were performed using a Hitachi SU8230 UHR Cold Field Emission SEM equipped with a Bruker X-Flash 5060FQ detector. A Raman microscope (LabRAM HR Evolution, Horiba Jobin Yvon) with a 633 nm laser was used for collecting the Raman spectra. CoPc/CNT was deposited on a titanium foil coated with 20 nm gold for surface-enhanced Raman scattering. Water contact angles were measured with the sessile drop method using a contact angle goniometer (OneAttension, Biolin Scientific). The interlayer spacings of CoPc/rGO/Nylon membrane were tested by X-ray diffraction (Rigaku SmartLab X-Ray Diffractometer).

Preparation of CoPc/CNT and CoPc/rGO

Received CNTs were calcinated at 500 ℃ in air for 5 hours. The calcinated CNTs were then sonicated in 5 wt.% aqueous HCl for 30 minutes, followed by stirring overnight. Finally, the CNTs were washed with deionized water until pH neutral and collected by freeze-drying to yield purified CNTs. 30 mg of purified CNTs in 30 mL of DMF and 1.5 mg of CoPc in 15 mL of DMF were prepared separately by sonication. The sonication usually took an hour to achieve good dispersion of CNTs and dissolution of CoPc. The CNT suspension and CoPc solution were then mixed and sonicated for another hour followed by stirring overnight at room temperature. Subsequently, the mixture was centrifuged. The precipitate was washed with DMF and centrifuged twice until the supernatant became colorless. The received precipitate was washed several times with deionized water to remove the remaining DMF. The washed precipitate was then freeze-dried to yield the hybrid material. The preparation of CoPc/rGO followed the same procedure as CoPc/CNT except that rGO was used in the place of CNTs. rGO was prepared by heating a 25 mL of 5 mg/mL GO/ethylene glycol (EG) solution at 180 ℃ for 24 hours in a 40 mL Teflon-lined stainless-steel autoclave. The GO/EG solution was obtained by sonicating 125 mg of GO nanosheets (CARMERY NEW MATERIAL CO., LTD) in 25 mL of EG for an hour.

Preparation of electrodes

2 mg of catalyst in 2 mL of ethanol containing 6 μL of a 5 wt.% Nafion solution (Sigma-Aldrich) was sonicated for 1 hour to prepare a catalyst ink. The prepared ink was drop-casted on a 3.0×1.0 cm^2 polytetrafluoroethylene-treated carbon fiber paper (Toray 030, Fuel Cells Store) and dried under an IR lamp. The covered geometric area was 1.0×1.0 cm^2, giving a catalyst loading of 0.4 mg/cm^2.

Electrochemical measurement

Electrochemical TCE dechlorination was carried out in purified 0.1 M $KHCO_3$ aqueous electrolyte at room temperature and under atmospheric pressure with a custom-designed gas-tight H-cell (12 mL of electrolyte and ~18 mL of gas headspace for each compartment). Ar was flown at 20 s.c.c.m. through a pure TCE liquid to carry TCE vapor into the cathodic compartment for 15 min before electrolysis. Ar with TCE vapor was kept bubbling into the electrolyte during electrolysis to maintain a saturated TCE concentration of ~9 mM. A graphite rod (purchased from Sigma-Aldrich) was used as the counter electrode, and an Ag/AgCl (saturated KCl) electrode (purchased from Pine Research Instrumentation) was used as the reference electrode. An anion-exchange membrane (Selemion DSV) separated the cathodic and anodic compartments. A Bio-Logic VMP3 multi-potentiostat was used for all the electrochemical experiments. Electrochemical impedance spectroscopy measurements between 200 kHz and 1 Hz with an amplitude of 10 mV were conducted to determine the ohmic drop between the working and reference electrodes at −0.5 V vs Ag/AgCl. In the Nyquist plot, the curve's intersection with the real axis was collected as the resistance, which was automatically corrected with 100% iR compensation during all the electrochemical measurements. The current was normalized to the catalyst-covered geometric area. The gas products were detected using an online gas chromatography system (MG #5, SRI Instruments) which injected 0.1 mL of the outlet gas every 15 min for measuring C_2H_4 and H_2 concentrations. At the end of each electrolysis, 0.45 mL of the electrolyte was taken out of the H-cell and mixed with 0.05 mL of D_2O in an NMR tube for ^{1}H NMR spectroscopy measurements with water suppression using a Bruker 400 MHz Broadband Probe. The partially dechlorinated products were screened by comparing their standard spectra to the sample spectrum (Fig.S2).

Preparation of CoPc/rGO@Nylon

CoPc/rGO was dispersed in a mixed solvent of ethylene glycol and DMF (1/10, v/v) under sonication to form a homogenous suspension (~0.42 mg/mL). The suspension was vacuum filtrated through 0.2 μm Nylon membranes (purchased from Cytiva) to prepare CoPc/rGO@Nylon membranes as illustrated in Fig.S12. The obtained wet membranes were washed with DI water several times and then kept in water before using. The effective area of each membrane was 9.6 cm^2 with a diameter of ~ 3.5 cm. In control experiments, CoPc/CNT@Nylon and rGO@Nylon membranes were prepared with the same method.

Electro-filtration measurement

A 3D-printed dead-end filtration cell (Fig.4a) was employed to evaluate the electro-filtration process. The cell was composed of a feed chamber, a permeate chamber, and two electrodes. A CoPc/rGO@Nylon membrane and a RuO_2-IrO_2/Ti mesh (purchased from TIBROMTACK Store) were used as the cathode and anode, respectively. An insulating silicone rubber gasket was applied to separate the two electrodes and seal the cell. The feed solution was deionized water containing 20 mM of Na_2SO_4 and ~1 mM of TCE unless otherwise stated. The feed chamber was filled with N_2 gas to a designated pressure in the range of 0.75 to 5 psi. An electrochemistry workstation (CH Instruments, 760E) was used to maintain a consistent current density between the electrodes. After operation (typically 30 minutes unless otherwise mentioned).

TCE in the feed or permeate solution was quantified by ^{1}H NMR spectroscopy with water suppression. 450 μL of the corresponding solution was mixed with 50 μL of D_2O containing10 mM dimethyl sulfoxide (DMSO) and 50 mM phenol as internal standards for the ^{1}H NMR analysis. The concentration of TCE was calculated using the ratio between the area of the TCE peak (at the chemical shift of 6.67 ppm) and that of the DMSO internal standard (at the chemical shift of 2.6 ppm).

The Cl^- concentration in the permeate solution was quantified using a HANNA Instruments 93753 Chloride ISM kit. For each measurement, 1 mL of permeate solution was collected and diluted to a final volume of 9 mL with deionized water in a 50 mL centrifuge tube. The diluted solution was then dosed with 0.5 mL of the HI 93753A-0 displacing reagent and mixed using a vortex mixer. After that, 0.5 mL of the HI 93753B-0 complexing reagent was added into the solution, followed by vortex mixing. The final solution was filled into a 1 cm × 1 cm × 3 cm glass cuvette for UV-vis measurements with a Shimadzu UV-2600 UV-vis spectrophotometer. The absorbance at 454 nm in the received spectrum was then fitted to a calibration curve obtained in the same way with standard NaCl solutions to determine the Cl^- concentration (Figure.S18).

Electro-filtration performance, including TCE rejection (R, %) and water flux (J_W, $L \cdot m^{-2} \cdot h^{-1}$) were calculated using the following equations:

$$R_{(\%)} = 100 \times \frac{C_f - C_p}{C_f}$$

$$J_W = \frac{V}{A \times t}$$

where C_f and C_p are TCE concentrations of the feed and permeate solutions, respectively, V is the permeate volume, A is the membrane effective area (9.6×10^{-4} m^2), and t is the operation time.

The decomposition percentage of the rejected TCE (D, %) was calculated using the following equation:

$$D_{(\%)} = 100 \times \frac{C_{Cl^-}}{3 \times (C_f - C_p)}$$

Where C_f and C_p are TCE concentrations of the feed and permeate solutions, respectively, C_{Cl^-} is the concentration of Cl^- in the permeate solution.

Energy consumption for TCE removal (E) was calculated by the following equation:

$$E = \frac{UI}{Q \log [\frac{C_p}{C_f}]}$$

where U is applied potential, I is current, Q is water flow rate, and C_f and C_p are TCE concentrations of the feed and permeate solutions, respectively.

Supplementary Figures

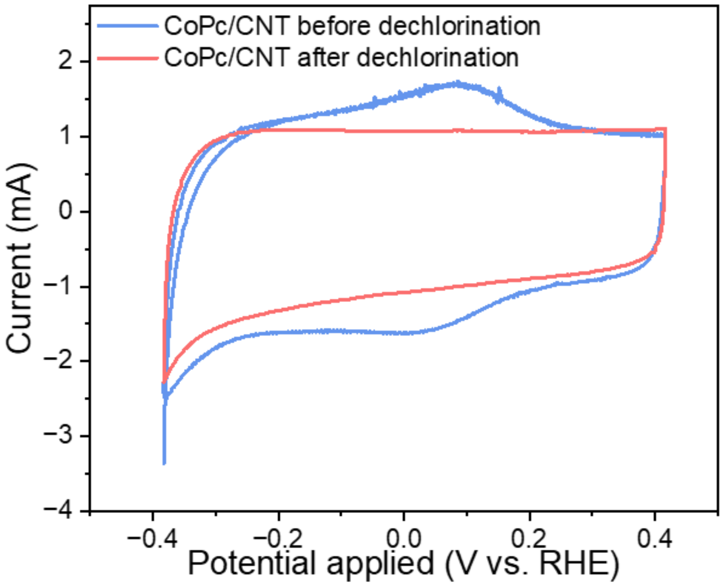

Figure S1. Cyclic voltammogram of pristine vs deactivated CoPc/CNT (after catalyzing TCE reduction in 0.1 M $KHCO_3$ aqueous electrolyte at -0.68 V for an hour) measured at 100 mV/s in Ar-saturated 0.1 M $KHCO_3$ aqueous electrolyte.

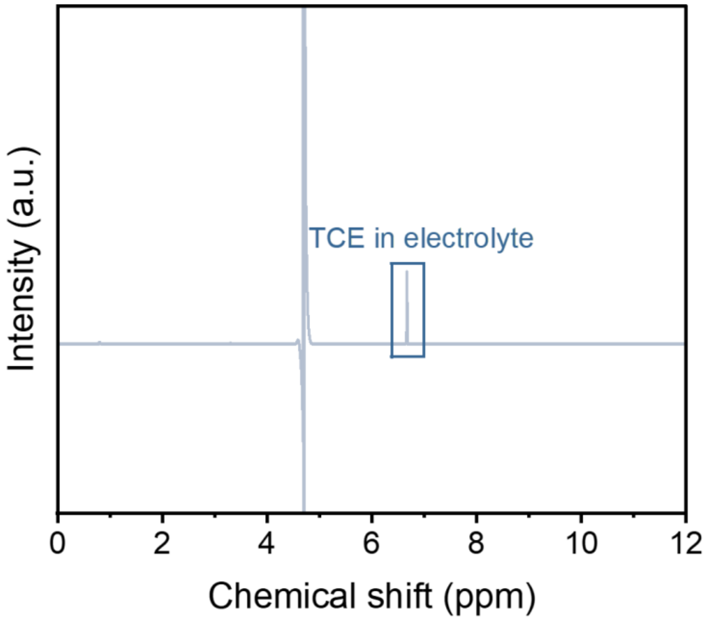

Figure S2. Typical ^{1}H NMR spectrum of electrolyte after TCE dechlorination catalyzed by CoPc/CNT.

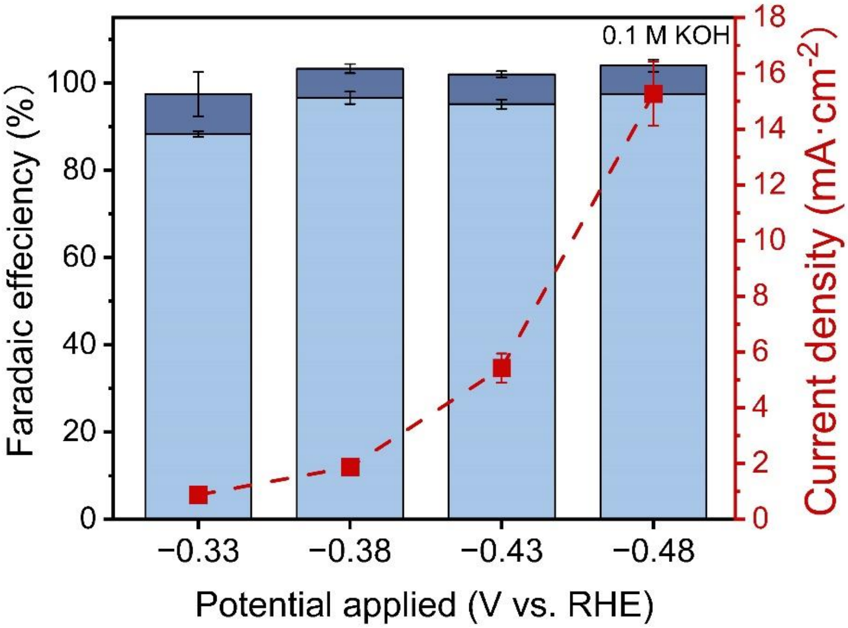

Figure S3. Electrochemical TCE dechlorination performance (FE and total current density) of CoPc/CNT in 0.1 M KOH aqueous solution saturated with TCE.

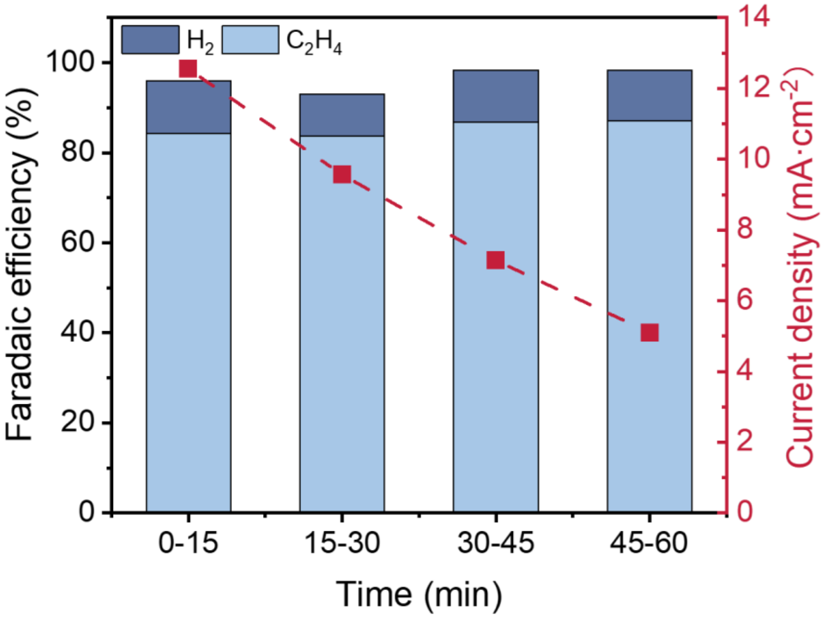

Figure S4. Stability of TCE dechlorination catalyzed by CoPc/CNT at -0.68 V in 0.1 M $KHCO_3$.

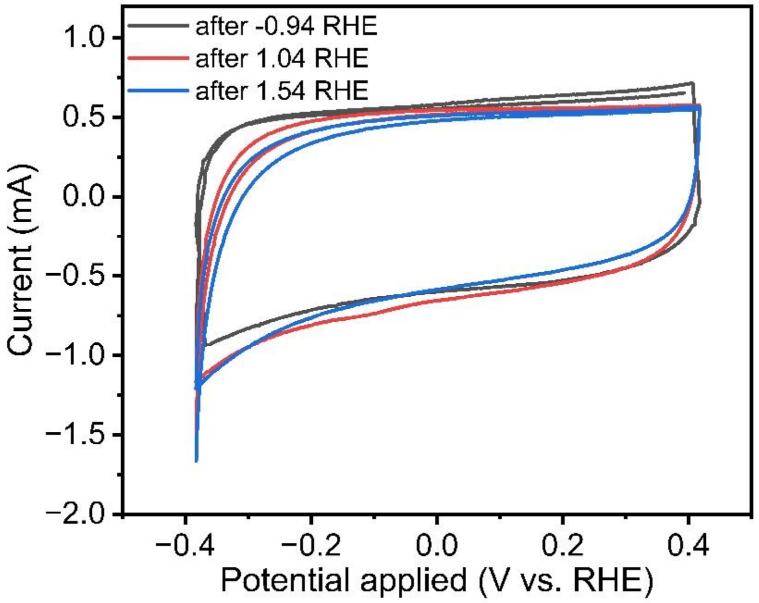

Figure S5. Cyclic voltammograms of deactivated CoPc/CNT (after catalyzing TCE reduction in 0.1 M $KHCO_3$ aqueous electrolyte at -0.68 V for an hour) after being held at various potentials for 15 minutes measured at 100 mV/s in Ar-saturated 0.1 M $KHCO_3$ electrolyte.

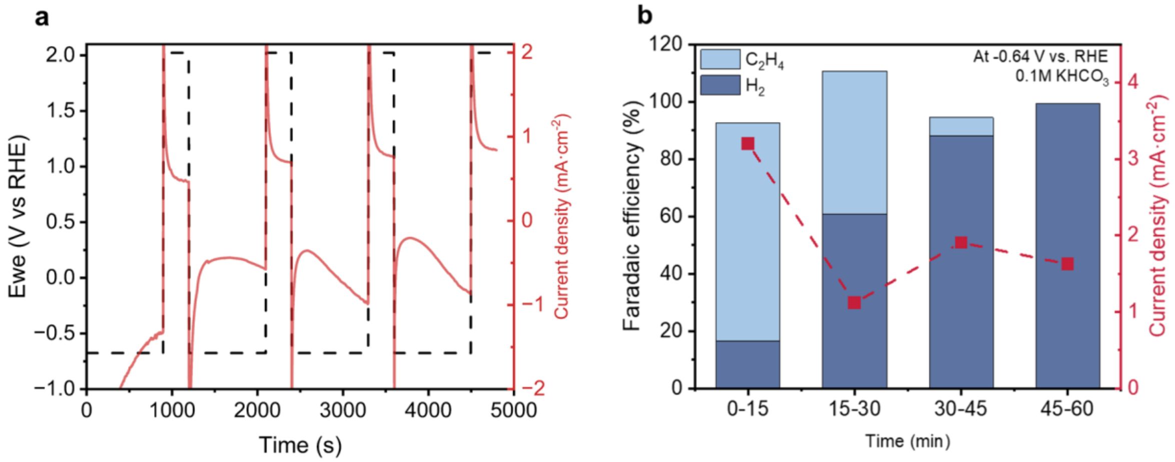

Figure S6. (a) Potential and current profiles and (b) total current density and FE of electrochemical TCE reduction catalyzed by CoPc/CNT in 0.1 M $KHCO_3$ aqueous electrolyte. The reduction potential was -0.64 V. Oxidative steps at 2.0 V were inserted in the hope of mitigating catalyst deactivation.

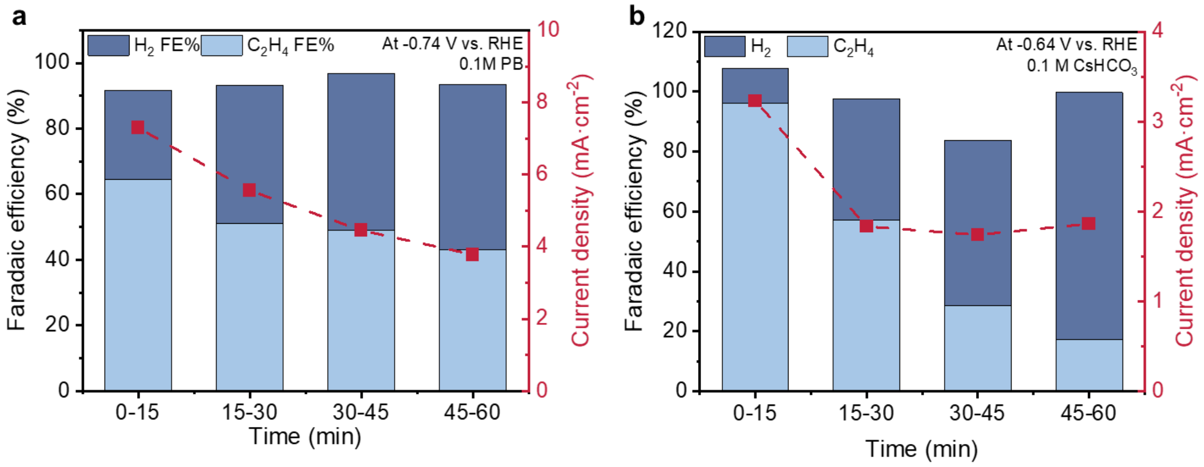

Figure S7. Electrochemical TCE reduction performance (total current density and Faradaic efficiency) of CoPc/CNT in (a) 0.1 M aqueous phosphate buffer (PB, pH = 7.2) at -0.74 V and (b) 0.1 M aqueous $CsHCO_3$ at -0.64 V.

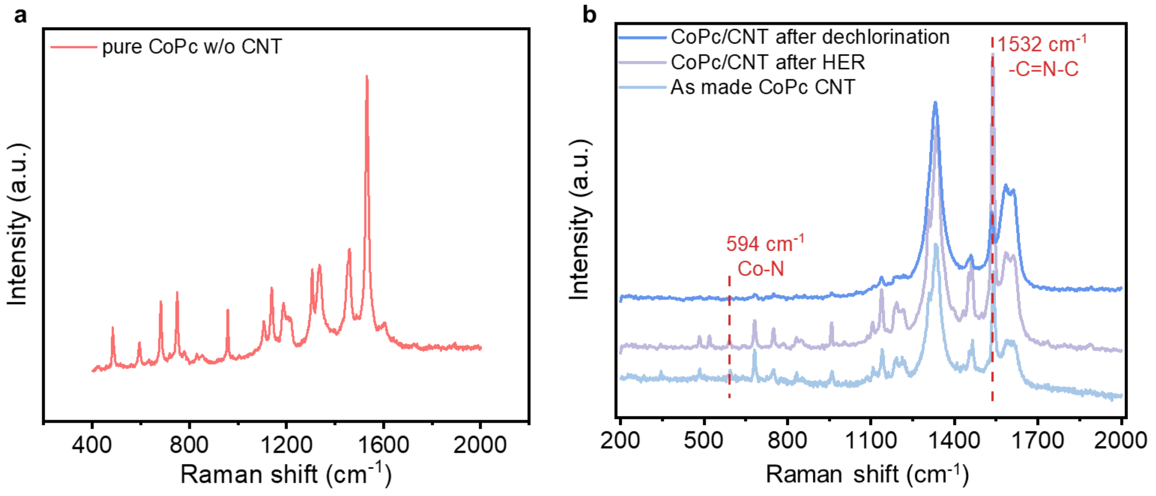

Figure S8. (a) Raman spectrum of pure CoPc powder. (b) Raman spectra of fresh CoP/CNT, CoPc/CNT after catalyzing HER, and CoPc/CNT after catalyzing TCE dechlorination in 0.1 M aqueous $KHCO_3$ at -0.64 V for an hour. Intensities were normalized to the D-band of CNT.

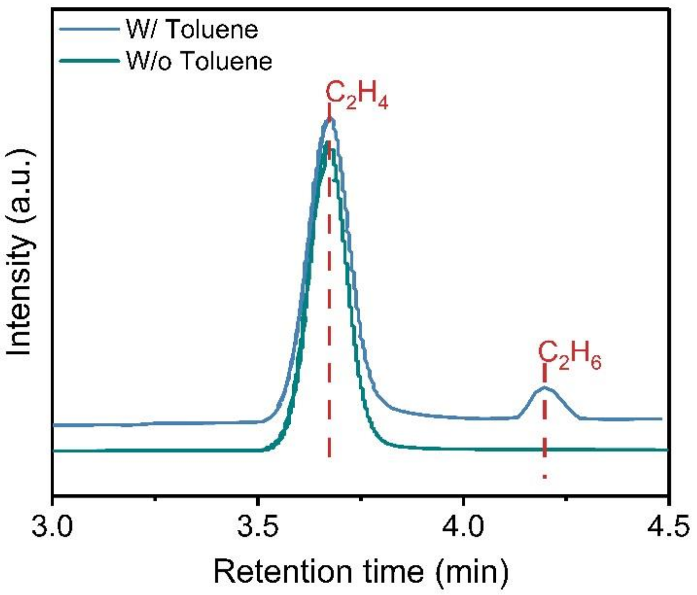

Figure S9. Gas chromatograph showing emergence of ethane from TCE dechlorination catalyzed by CoPc/CNT in the presence of toluene.

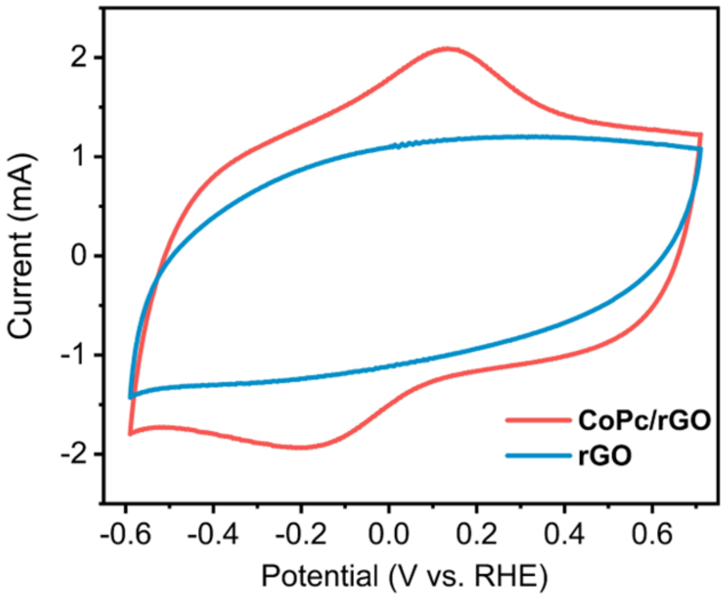

Figure S10. Cyclic voltammograms of CoPc/rGO and rGO measured at 100 mV/s in Ar-saturated 0.1 M $KHCO_3$ aqueous electrolyte.

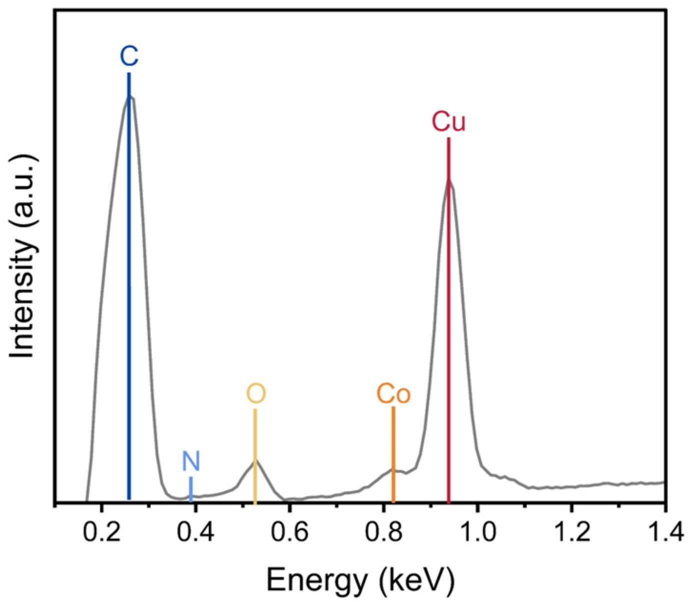

Figure S11. Representative EDS spectrum of CoPc/rGO (Cu signal was from the substrate).

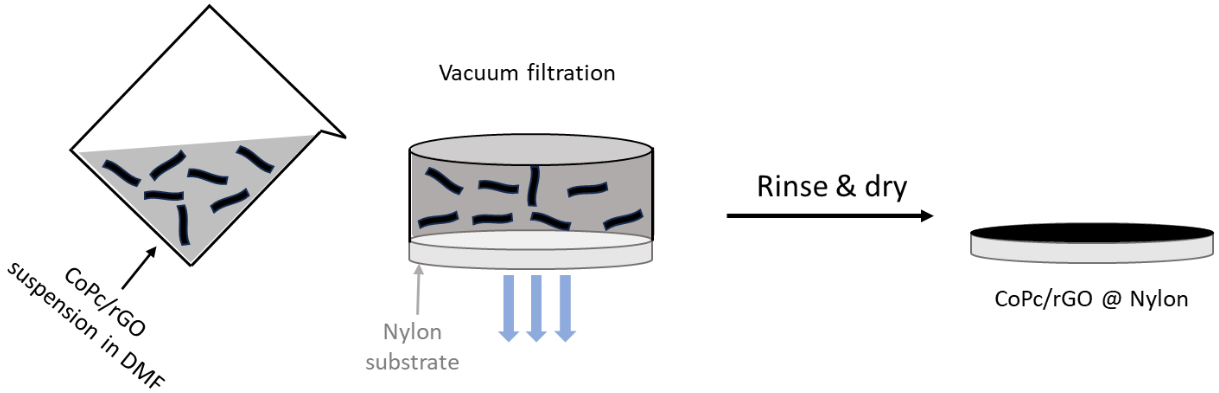

Figure S12. Schematic illustration of preparation of CoPc/rGO@Nylon membrane.

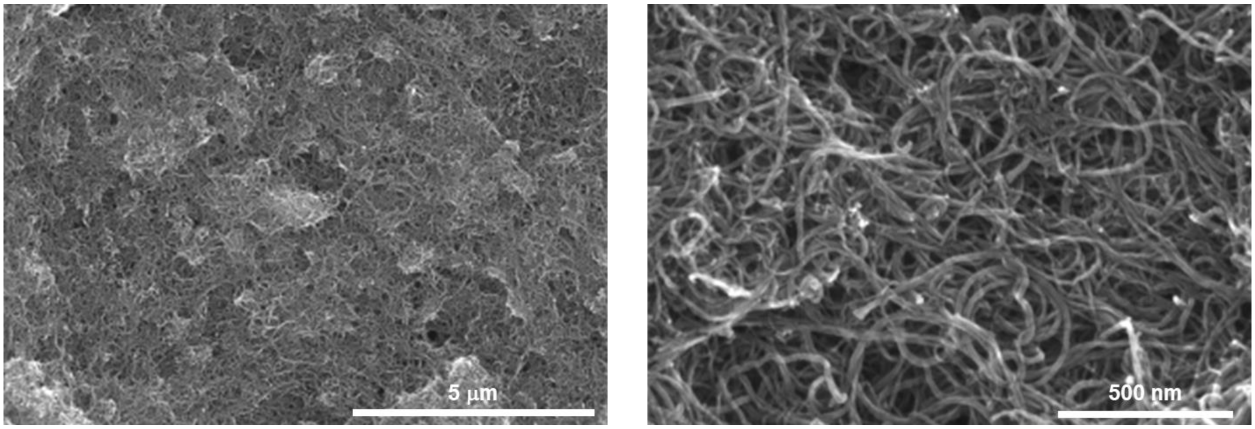

Figure S13. SEM images of CoPc/CNT loaded on Nylon membrane for electro-filtration.

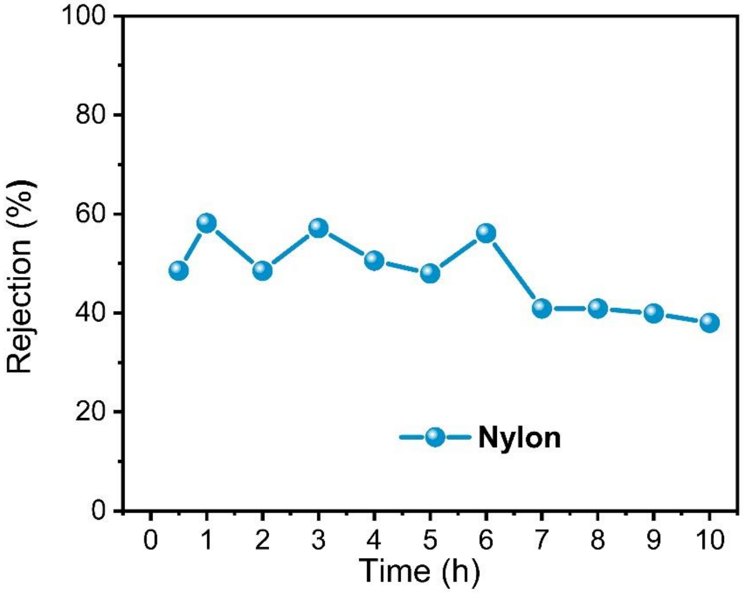

Figure S14. TCE rejection performance of bare Nylon membrane with a 1.25 psi transmembrane pressure and no potential applied.

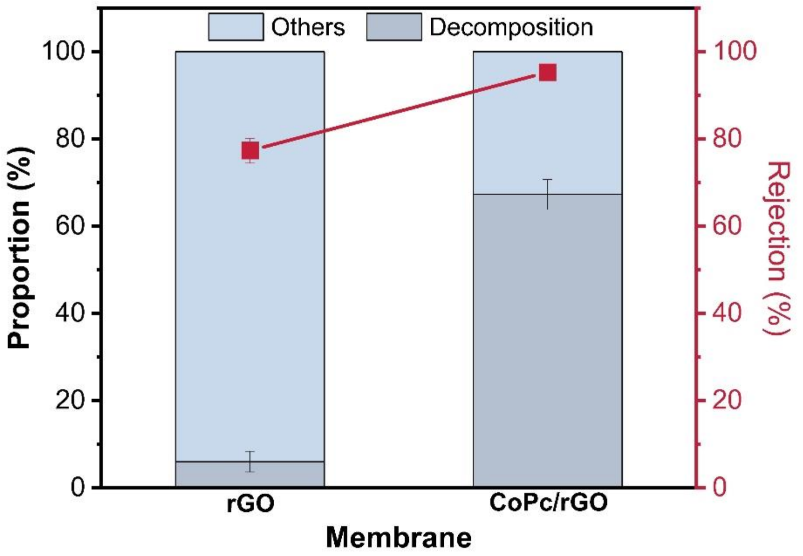

Figure S15. TCE rejection rate and decomposition proportion of rGO@Nylon vs CoPc/rGO@Nylon operating at current density of 2 mA·cm^{-2}, transmembrane pressures of 1.25 psi, and catalyst loading of 0.31 mg·cm^{-2}.

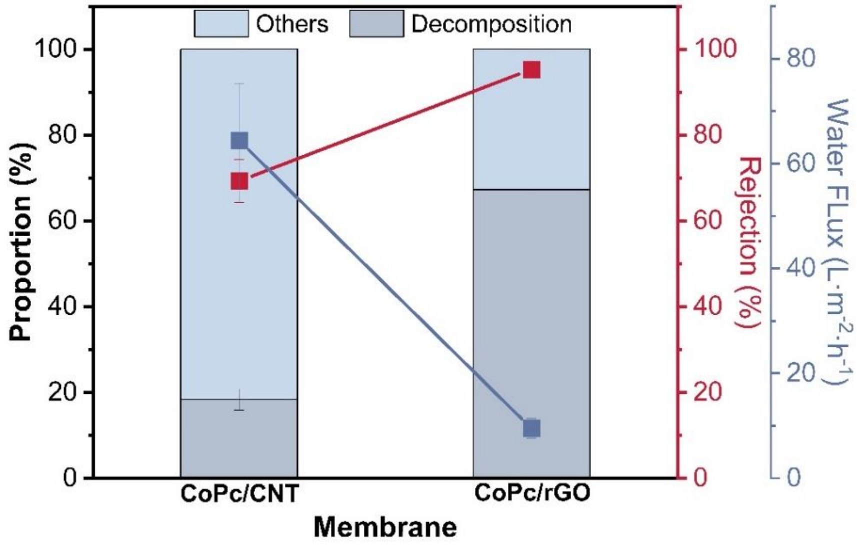

Figure S16. TCE rejection rate and decomposition proportion as well as water flux of CoPc/CNT@Nylon vs CoPc/rGO@Nylon at current density of 2 mA·cm^{-2}, transmembrane pressures of 1.25 psi, and catalyst loading of 0.31 mg·cm^{-2}.

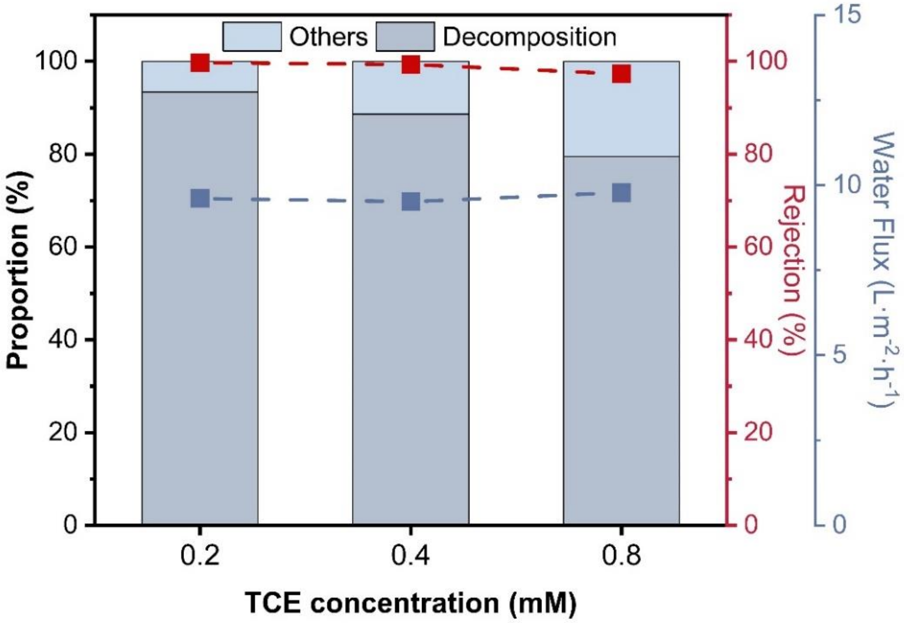

Figure S17. Electro-filtration performance (rejection rate, decomposition proportion, and water flux) of CoPc/rGO@Nylon with varying TCE concentrations at current density of 2 mA·cm^{-2}, transmembrane pressures of 1.25 psi, and catalyst loading of 0.31 mg·cm^{-2}.

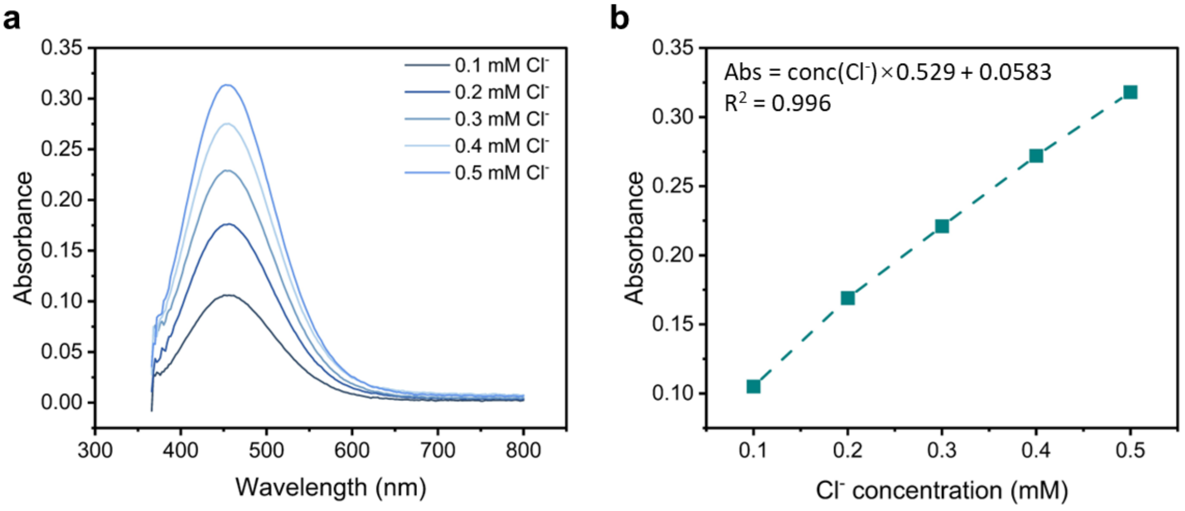

Figure S18. (a) UV-vis absorption spectra of standard sodium chloride solutions and (b) derived calibration curve for determining Cl$^-$ concentrations of sample solutions.
